# Coarse-Grained Modeling and Interpretation of Phenomenological Creep Rate Behavior with Experimental Validation

**DOI:** 10.3390/e28050482

**Published:** 2026-04-22

**Authors:** Tianci Gong, Daoqing Zhou, Xuefei Guan, Yi-Mu Du

**Affiliations:** 1 Graduate School of China Academy of Engineering Physics, Beijing 100193, Chinaxfguan@gscaep.ac.cn (X.G.); 2 China North Engine Research Institute, Tianjin 300400, China

**Keywords:** creep damage, creep rate, hierarchical modelling, bathtub-shaped curve, statistical physics

## Abstract

Creep is one of the main failure mechanisms of materials at elevated temperatures, and the creep rate curve is a key descriptor of creep deformation and damage evolution. However, existing creep models are mainly phenomenological or stage-wise, and the physical origin of the bathtub-shaped creep rate curve over the full creep process has not been systematically clarified. In this study, creep damage is treated as an aging failure process of a material system, and a physically interpretable hierarchical model is established based on statistical physics for disordered complex systems. By linking the evolution and interaction of microscopic material units with macroscopic creep behavior, the proposed model provides a unified description of the primary, secondary, and tertiary creep stages and offers a theoretical explanation for the bathtub-shaped creep rate curve. Validation using representative metallic and composite material cases shows that the model can reasonably reproduce the overall three-stage creep rate evolution, with residual sums of squares of 1.3088 and 0.5369, respectively. These results demonstrate the ability of the model to capture full-process creep behavior in different material systems. The main advantage of the proposed approach is its physical interpretability within a unified framework, while its current limitation is that the validation remains limited in scale and broader benchmark comparisons with conventional methods are still needed. This work provides a statistical perspective for creep behavior modeling and for understanding the microscopic mechanisms and interactions underlying creep degradation in structural materials.

## 1. Introduction

**Figure 1:**
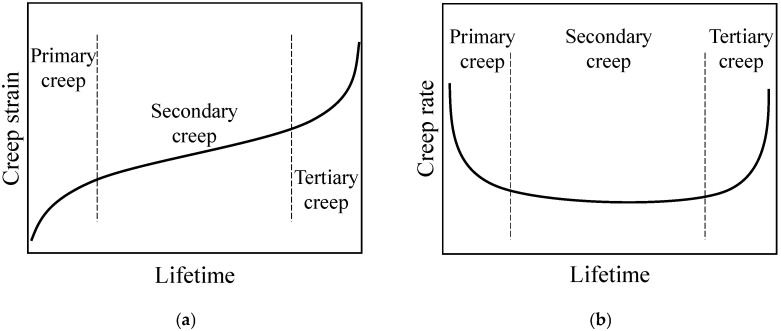
Three phases of creep process: (**a**) creep strain and (**b**) corresponding creep (strain) rate.

Creep is the one of the main causes of material and structural fractures at elevated temperature. The creep strain rate can phenomenologically describe the creep behavior of materials and becomes a fundamental approach for engineering predictions of creep damage and failure [1,2,3,4,5,6]. In practice, most observed creep processes consist of three stages, namely, primary creep, secondary creep, and tertiary creep, as shown in Figure 1a [7,8,9]. The corresponding creep rate curves of the three stages collectively exhibit a distinct bathtub-shaped curve, as illustrated in Figure 1b.

The modeling of creep behavior can be carried out at different scales [10]. Empirical modeling methods have been proposed to describe the relationship between the minimum creep rate and time to fracture based on creep testing data, such as the Monkman–Grant relationship, Larson–Miller parameter, and their variants [11,12,13,14]. Materials science models, which characterize creep rates and damage accumulation, can be constructed using parameters related to material microstructure and processing conditions, such as grain size, cavity diffusion, and dislocation migration. Common modeling variables include dislocation density [15], internal stress [16], and damage parameters [17]. In addition, micromechanical models based on representative volume elements can simplify the complicated microscale interactions among different microstructural features and can be used in conjunction with the finite element methods [18,19]. Existing studies have made important progress in describing creep deformation and damage evolution over different stages. Classical stage-based descriptions distinguish primary, secondary, and tertiary creep, where the secondary stage is often represented by Norton-type power-law formulations [20], while tertiary acceleration is commonly incorporated through damage mechanics models such as the Kachanov–Rabotnov (KR) framework and its modified forms [21,22]. For example, modified KR-type models have been used to evaluate creep–fatigue interaction and turbine-blade life, but some key parameters remain essentially empirical and do not have clearly identifiable microscopic physical meanings [23]. More recently, thermodynamically consistent creep models have attempted to incorporate mechanism-related damage contributions, such as cavity evolution and dislocation activity [24]. Such formulations focus mainly on the evolution from the onset of the minimum creep rate stage and provide a relatively weak description of the initial transient creep regime. Therefore, despite these advances, there remains a need for a framework that can describe the full primary–secondary–tertiary creep evolution while retaining clearer physical interpretability through an explicit micro-to-macro linkage.

In these models, the emergence of the bathtub-shaped curve as a whole has not yet been systematically explained from the perspective of the underlying physics, which renders this curve largely a phenomenological and engineering observation and has led to criticism and controversy regarding its rationality [25,26,27]. On the one hand, phenomenological data-driven methods are often constrained by economic cost and testing time, and the precise description of the entire creep process using only a few parameters can be highly nontrivial [28]. Such methods usually offer limited theoretical explanations for creep damage. On the other hand, structural materials contain complex microstructural features, such as dislocation structures, grain boundaries, and precipitates, whose mechanisms and interactions are difficult to quantify and describe accurately [10]. Moreover, many established models focus mainly on one or several stages of creep, for example, the primary or secondary creeps, and the full-stage creep process is often represented as a combination of segmented descriptions. Therefore, an open question remains as to whether the phenomenological bathtub-shaped creep rate curve can be modeled and interpreted using a framework developed from more fundamental physical principles. In recent years, efficient approximation and probabilistic modeling techniques have also been actively developed for complex engineering systems, including uncertainty quantification of black-box models, quasi-real-time reliability assessment, and structural response reconstruction based on experimental approximation and model reduction [29,30,31,32,33]. These studies suggest that interpretable reduced-order or probabilistic representations can provide useful insights into complex macroscopic behaviors induced by many underlying microscopic mechanisms.

As noted in Ref. [34], cavity density plays a crucial role in explaining the microscale creep damage behavior. The cavity density evolves through the nucleation and growth of cavities during creep; therefore, its dynamical evolution has been used to describe various damage phenomena at different scales and locations in materials [35,36]. Meanwhile, advanced characterization and imaging techniques have also been developed for flaw detection and size quantification in engineering components with complex geometries, providing useful support for the identification and interpretation of damage evolution in structural materials [37]. Since plastic deformation is closely related to material damage, it is reasonable to consider cavity density as a microscopic state variable associated with material failure. Recently, the failure-rate function of a composite system has been modeled by cascading component failure rates through a hierarchical structure function. A hierarchical structure in this context loosely refers to a system involving multiple components at different levels, following a prescribed structure function [38,39]. It has been shown that the statistically observed failure-rate behavior of a system involving many components can be less sensitive to the failure-rate behavior of an individual component. Moreover, when the number of levels and components is sufficiently large, certain features of the failure rate of the whole system can emerge regardless of the specific features of the individual components [40]. Thus, the macroscopic behavior resembles an emergent effect of complex systems, that is, a large number of microscale, component-level behaviors can collectively characterize macroscale, system-level regularities, while the system-level behavior becomes less sensitive to the details at the component level [41].

To address the above question, this study investigates the evolution of cavity density during the creep process from the perspective of coarse-grained modeling. The focus of the present work differs from existing creep studies in that the material is treated as a hypothetical system composed of numerous microscopic material volumes, whose states are governed by failure rates associated with cavity evolution. Based on this viewpoint, a hierarchical framework is established to describe the cascading relationships among microscopic volumes under creep driving forces and to connect these microscopic damage processes with the macroscopic creep rate response of the material specimen. In this sense, the creep rate curve is modeled in analogy with the failure-rate curve in reliability theory [42,43]. Through the proposed coarse-grained hierarchical framework, this study aims to interpret phenomenological creep rate behavior from a statistical-physics perspective by linking microscopic cavity evolution to the macroscopic full-stage creep response. The method is intended for the unified modeling of primary, secondary, and tertiary creep at elevated temperature and provides a more physically interpretable alternative to conventional phenomenological fitting or segmented stage-wise descriptions. The main novelty of the present work is that it offers a hierarchical mechanism-based explanation for the bathtub-shaped creep rate curve, thereby contributing both a new modeling framework and a new physical interpretation of this long-observed behavior. More specifically, the physical insight provided by the present approach is that the bathtub-shaped creep rate curve is interpreted as the collective statistical manifestation of cavity-related microscopic damage evolution, rather than only as a phenomenological macroscopic trend. Compared with conventional creep damage theories that mainly describe damage evolution through macroscopic variables or treat different creep stages separately, the proposed framework makes explicit the micro-to-macro linkage between cavity evolution and the full-stage creep response within a unified hierarchical picture.

The remainder of this study is organized as follows. Section 2 describes the basic physical quantities in the creep hierarchical modeling process and models the creep damage process from microscales to macroscales, followed by parametric studies on key parameters of the model. Section 3 discusses the theoretical threshold for the emergence of bathtub-shaped creep rate curves in detail. Section 4 demonstrates and verifies the proposed method using realistic creep testing data. It is shown that the developed method can capture the whole creep process reliably.

## 2. Hierarchical Modeling of Creep Damage Process

Cavity density is a commonly used microscopic parameter for material state characterization in the creep process. In practice, it can be quantified by the number or the size of cavities per unit area or volume [44], or by the rate of change in volume per unit weight [45]. In this study, the concept of cavity density is adopted following Refs. [45,46], and is used as a parameter to characterize the microscopic degradation behavior in the modeling. Following these conventions, the cavity density in this study is defined as the fraction of the total area/volume of cavities in a given area/volume, i.e., a percentage value, denoted by $\rho_{c}\left(t\right)\in [0,1]$. The variation in the cavity density can thus be correlated with the state of the material as a measure of damage.

### 2.1. Microscopic Mechanisms and Behavior of Creep Damage

For the convenience of algebraic manipulations, an auxiliary state variable $\rho_{s}(t)$ can be defined as $\rho_{s}(t)=1-\rho_{c}(t)$, representing the remaining pristine portion of the material. The cavity density rate, which measures the change in the cavity density in a finite time interval, can be expressed using $\rho_{s}\left(t\right)$ as

(1) $$-\frac{\Delta \rho_{s}\left(t\right)}{\Delta t}=\frac{\rho_{s}\left(t\right)-\rho_{s}\left(t+\Delta t\right)}{\Delta t}.$$

Bowring et al. [46] and Miller and Langdon [45] have noted that the quantity, $\;-\Delta \rho_{s}(t)/\rho_{s}(t)$, relates to the creep strain ϵ via

(2) $$-\frac{\Delta \rho_{s}\left(t\right)}{\rho_{s}\left(t\right)}=\frac{\rho_{s}\left(t\right)-\rho_{s}\left(t+\Delta t\right)}{\rho_{s}\left(t\right)}\propto \epsilon .$$

The creep strain rate can thus be expressed as

(3) $$\dot{\epsilon }\approx \frac{\Delta \epsilon }{\Delta t}\propto -\frac{\Delta \rho_{s}\left(t\right)}{\Delta t}\frac{1}{\rho_{s}\left(t\right)}\;.$$

Therefore, by modeling the cavity density variation over time, it provides a means of characterizing the macroscopic creep rate. Next, micro-mechanisms of the dynamical evolution of the cavity density in the creep process are discussed and mathematical models are formulated.

Kassner and Hayes [34] investigated the dynamical evolution of cavities, including nucleation and growth during creep. In this study, two main mechanisms underpinning the dynamical behavior of the cavity, namely cavity accumulation and dislocation pile-up, are focused on. Raj and Ashby [47] noticed the phenomenon that cavities gradually accumulate over time and pointed out that the cavity accumulation promotes the generation of new cavities. The generation is influenced by the cavity density and can be more active at grain boundaries. Dislocation pile-up is a process in which dislocations glide and gradually aggregate over time to form dislocation blocks [48]. The migration of dislocations is affected by dislocation pile-up; therefore, the variation in dislocation density can in certain degree reflect the change in cavity density caused by dislocation pile-up. Dislocation pile-up promotes the nucleation of cavities, but during the early stages of creep, dislocations tend to migrate towards grain boundaries, resulting in a decrease in dislocation density within grains [49,50]. Dislocation pile-up mainly occurs at grain boundaries, and cavities can also form within grain due to dislocation pile-up, but the growth of cavities within grains is relatively slow, resulting in a lower cavity density than that at grain boundaries [34]. Based on the above mechanisms observed in experimental investigations, it is known that the cavity accumulation and the dislocation pile-up can have different effect on the dynamical evolution of cavities, depending on the actual locations of the two driving factors. Therefore, the change in the cavity density caused by the two mechanisms at grain boundaries and within grains needs to be considered separately. To characterize the time-dependent variation in the cavity density $\rho_{c}(t)$, a time increment variable t is introduced, allowing for analyzing the instantaneous behavior of the cavity density using $\rho_{c}(t)$ and $\rho_{c}\left(t+t\right)$.

**Figure 2:**
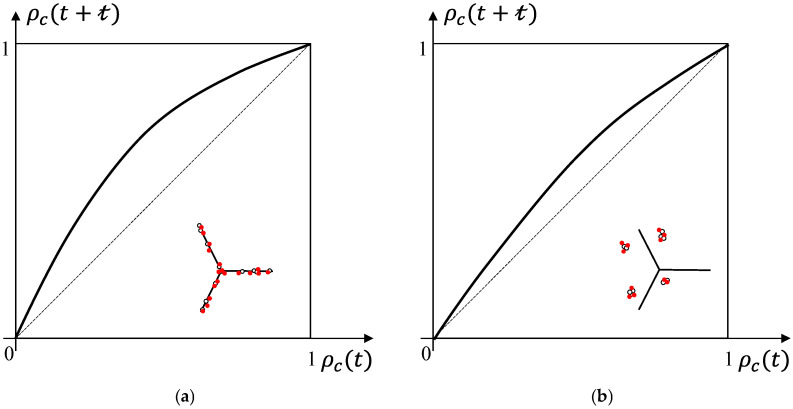

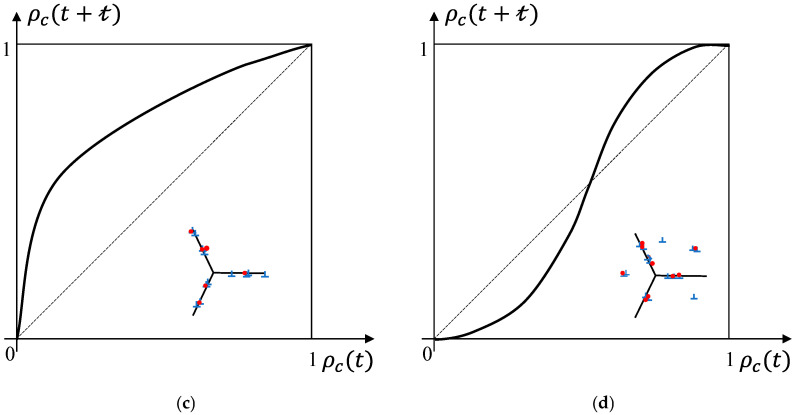
Variation in the cavity density caused by cavity accumulation (**a**) at grain boundaries and (**b**) within grain, and by dislocation pile-up (**c**) at grain boundaries and (**d**) within grains.

Figure 2 shows the four possible relationships between the cavity densities $\rho_{c}(t)$ and $\rho_{c}(t+t)$, owing to the collective effects of the two mechanisms at grain boundaries and within grains. Figure 2a,b illustrate the changes in cavity densities caused by cavity accumulation, where black hollow dots and red solid dots (in the inset) represent the original cavities and nucleation of new cavities caused by cavity accumulation, respectively, and the triad segment (in the inset) represents grain boundaries. As the cavity density increases, the effect of cavity accumulation gradually strengthens, leading to a significant increase in cavity density due to cavity accumulation. As the cavity density becomes large, the space for cavity nucleation is restricted and the effect of cavity accumulation weakens, resulting in a gradual decrease in cavity density. Consequently, the curves of $\rho_{c}(t+t)$ vs. $\rho_{c}(t)$ under the mechanism of cavity accumulation are convex in both Figure 2a,b. Moreover, as cavity nucleation often occurs at grain boundaries, the effect of cavity accumulation at grain boundaries is stronger than that within grains [34]. Such a difference is signified by different curvatures of the two curves shown in Figure 2a,b.

Figure 2c,d illustrate two different scenarios of the cavity density evolution promoted by dislocation pile-up. In the inset, the blue ⊥ represents dislocations, the overlapping of multiple dislocations indicates dislocation pile-up, and the red dots represent the cavities nucleation induced by dislocation pile-up. In the early stage of the primary creep, dislocations first migrate towards grain boundaries. The migration has two consequences. In the vicinity of grain boundaries, the dislocations increase as more dislocations within grains move to the region. As a result, an aggravation of dislocation pile-up occurs. Due to the dislocation pile-up, strain hardening becomes dominant, which slows down the increase in dislocation density in the region [49]. As the dislocation pile-up promotes nucleation and growth of cavities in this case, the cavity density has the same trend of variation as the dislocation density. Therefore, for the cavity density at grain boundaries, the cavity density curve presents as a convex curve as shown in Figure 2c. On the contrary, for dislocations within grains, they first migrate towards grain boundaries, leading to a reduction in the dislocation density within grains. Once these existing dislocations approach grain boundaries, new dislocations develop and promote new cavities until saturation [51]. This implies that the cavity density within grains reduces at the beginning then increases afterwards, as shown in Figure 2d.

**Figure 3:**
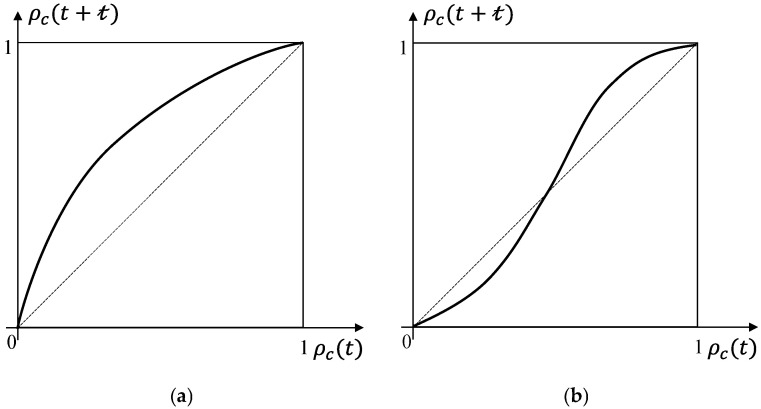
Two types of cavity density variation curves: (**a**) at grain boundaries and (**b**) within grains.

In summary, the two mechanisms, cavity accumulation and dislocation pile-up, collectively act at grain boundaries and within grains, resulting in two types of cavity density variation patterns in the creep process as illustrated in Figure 3. The two types of cavity density variations are used to describe the damage state of a microscopic volume. By cascading these microscopic volumes gradually, the hierarchical model of the hypothetical system representing the material specimen as a whole is developed.

### 2.2. Hierarchical Model of the Creep Damage Process

Considering the creep process as the material damage process, hierarchical modeling from microscale to macroscale can be performed by hierarchical discretization of the material specimen, followed by the incorporation of a proper structure function.

#### 2.2.1. Hierarchical Discretization

**Figure 4:**
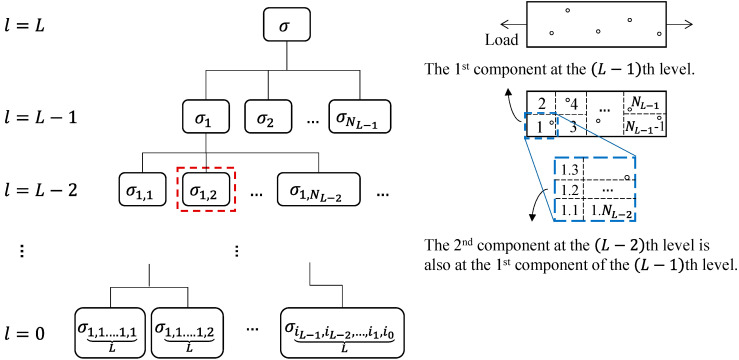
Schematic diagram of the hierarchical system model for creep process.

A hierarchical system loosely refers to a model consisting of multiple levels, in which structure functions are used to describe the relationships between different levels. Creep is considered as a material damage process, and the creep degradation behavior can be modeled by utilizing microscopic degradation parameters. Consider a hierarchical system of L+1 levels, denoted by l=0,1,…,L, where l=0 and l=L indicate the microscopic level and macroscopic level. The number of levels L determines the level of refinement of the model. Figure 4 shows a schematic diagram of the hierarchical system of the creep process, in which the state of a higher level is modeled by the states of lower levels via structure functions.

To avoid confusion, any state variable $\sigma_{i}$ within the hierarchical system is labeled by the subscript i, where the subscript $i=\left(i_{L-1},i_{L-2},\ldots ,i_{l}\right)$ and $i_{l}$ represents the ith component at the lth level. Subscript i labels and the internal components within the system by recording the component indexes from the system level to the lth level. For instance, the states $\sigma_{1}$ and $\sigma_{2}$ represent the states of the first and second components at the (L−1)th level, respectively. $N_{L-1}$ in Figure 4 represents the total number of components at the (L−1)th level, and $\sigma_{N_{L-1}}$ represents the state of the last component at the (L−1)th level. The state at the lth level is denoted as $\sigma_{\left(i_{L-1},i_{L-2},\ldots ,i_{l}\right)}$, where the subscript records the component indexes from the system level to the lth level. The state of the lowest level is represented as $\sigma_{\left(i_{L-1},i_{L-2},\ldots ,i_{l+1},i_{l},\ldots ,i_{1},i_{0}\right)}$. For example, the $\sigma_{1,2}$ marked by a red square represents the state of being in the second component of the (L−2)th level and in the first component of the (L−1)th level. The state of higher-level components in a hierarchical system is determined by the states of lower-level components based on the structure functions. Therefore, the state of the (l+1)th-level component depends on all the states of the lth-level component, which is represented as

(4) $$\sigma_{\left(i_{L-1},i_{L-2}\ldots ,i_{l+1}\right)}^{\left(l+1\right)}=\phi^{\left(l+1\right)}\left(\sigma_{\left(i_{L-1},i_{L-2}\ldots ,i_{l+1},1\right)}^{\left(l\right)},\sigma_{\left(i_{L-1},i_{L-2}\ldots ,i_{l+1},2\right)}^{\left(l\right)},\sigma_{\left(i_{L-1},i_{L-2}\ldots ,i_{l+1},3\right)}^{\left(l\right)},\ldots \right),$$

where superscript (l) is used to indicate different levels, and $\phi^{\left(l+1\right)}$ is the structure function (a Boolean function) of the (l+1)th level. The cavity density, as a parameter characterizing material degradation, can be used to define the state of the system and components. Given the failure threshold defined by a specific cavity density ${\bar{\rho }}_{c}$, the state of the system and the components has two possible states {1,0}, where $\sigma_{i}=1$ represents the survival state and $\sigma_{i}=0$ denotes the failed state. Supposing the state of individual components is a random variable independently and identically distributed, then $\rho \equiv \mathrm{Prob}\left(\sigma_{i}=1\right)=\rho_{s}$. $\rho_{s}^{\left(l\right)}(t)$ is the survival probability (one minus cavity ratio) of any components at level l at time t. For simplification, the notation (t) is omitted. The survival probability of the (l+1)th level can be expressed using the set for the survival probability of all components at the lth level as

(5) $$\rho^{\left(l+1\right)}=f_{\phi^{\left(l+1\right)}}\left(\rho^{\left(l\right)}\right),$$

where the survival probability of each component at same level are independently and identically thus the lower index i is omitted. To facilitate the expression of iterative operations, the function operator ∘ is adopted such that f∘g(x)≡f(g(x)). The survival probability $\rho^{\left(l\right)}(t)$ at the lth level can be represented as

(6) $$\rho^{\left(l\right)}=f_{\phi^{\left(l\right)}}\circ f_{\phi^{\left(l-1\right)}}\circ f_{\phi^{\left(l-2\right)}}\circ \ldots \circ f_{\phi^{\left(1\right)}}\left(\rho^{\left(0\right)}\right),$$

where $\phi^{\left(l\right)}$ represents the structure function at the lth level. The survival probability of an L-level system is

(7) $$\rho^{\left(L\right)}=f_{\phi^{\left(L\right)}}\circ f_{\phi^{\left(L-1\right)}}\circ f_{\phi^{\left(L-2\right)}}\circ \ldots \circ f_{\phi^{\left(1\right)}}\left(\rho^{\left(0\right)}\right).$$

The material is considered a system, and the components are described using the different regions in the actual material system. Using the microscopic state parameters and structure functions, it allows us to model the creep degradation behavior in a bottom-up manner.

The construction of a creep hierarchical system needs to determine the specific standard for hierarchical division, the number of hierarchical levels from microscale to macroscale, and the corresponding regions for each level. From the perspective of statistical physics, size (length/area/volume) can be used as a standard for hierarchical division in the creep damage process, and smaller size indicates lower levels, whose relationship between level and region is described on the right side of Figure 4. The number of hierarchical levels and the corresponding regions for different levels should be provided based on engineering practicality considering the cost and accuracy of the modeling method. To perform hierarchical modeling of the creep damage process, further determination of microscopic state information and structure functions is required. Microscopic state information can be determined based on the statistical methods and cavity density evolution data, which can be collected through experimental methods provided in Refs. [44,46,52].

#### 2.2.2. Structure Function

**Figure 5:**
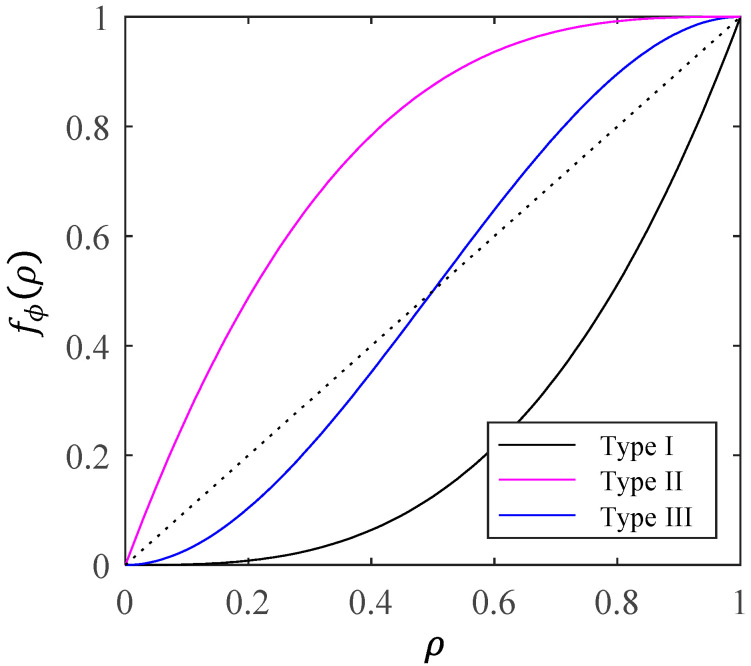
Illustration of different types of structure functions.

The structure function is a mathematical expression describing the mapping of survival probabilities between the adjacent hierarchical levels. In the proposed creep hierarchical modeling method, the structure function $\phi^{\left(l+1\right)}$ describes the relationship between the survival probabilities $\rho^{\left(l+1\right)}(t)$ and $\rho^{\left(l\right)}(t)$ of any lth level as shown in Equation (5). The initial survival probability is equal to 1, indicating that the material is undamaged. When the material completely fails, theoretically both the macroscopic and microscopic survival probability approach 0. Therefore, the structure function ϕ is a monotonically decreasing function during the creep damage process. Equation (5) points out that the survival probability of the (l+1)th level is determined by the set for the survival probability of all components at the lth level $\rho^{\left(l\right)}\;$based on the structure function. The survival probability functions at the same level l are assumed to be independently and identically distributed [40]. Given a set of non-negative integers $a_{j}=1,2,\ldots ,n$, the structure function of any (l+1)th level is expressed as

(8) $$f_{\phi^{\left(l+1\right)}}\left(\rho^{\left(l\right)}\left(t\right)\right)=\sum_{j=0}^{N_{l}}{a_{j}\left(1-\rho^{\left(l\right)}\left(t\right)\right)}^{N_{l}-j}{\left(\rho^{\left(l\right)}\left(t\right)\right)}^{j},$$

where $N_{l}$ is the total number of components at any lth level. Superscripts (l), subscripts i, and notation (t) are omitted for ease of presentation. $f_{\phi }\left(\rho \right)$ and ρ represent the survival probability of any lth level and the modeling results determined by the increasing Boolean function ϕ, respectively. The modeling results are survival probabilities at higher hierarchical levels. If referring to the survival probability of a specific layer, it is labeled by superscript (l). The structure function is presented in Figure 5 and can be categorized into three types [25], which are:

Type I: $f_{\phi }\left(\rho \right)<\rho$,

Type II: $f_{\phi }\left(\rho \right)>\rho$,

Type III: the formula $f_{\phi }\left(\rho \right)=\rho$ has a unique solution with ρ∈(0,1), which is $\rho =\rho^{*}$. When $\rho >\rho^{*}$, $f_{\phi }\left(\rho \right)>\rho$. When $\rho <\rho^{*}$, $f_{\phi }\left(\rho \right)<\rho$.

#### 2.2.3. Coarse-Graining Procedure

In the proposed creep hierarchical modeling framework, determining the structure functions requires a consistent identification of the effective dynamical rules at the grain scale. We therefore clarify the relation between the structure function types and Figure 3.

The transformation relations shown in Figure 3a,b should be interpreted as local temporal update operators acting at the grain scale, rather than as spatial coarse-graining operations. They define the effective evolution laws of the cavity fraction for two elementary microstructural constituents: grain-boundary regions and grain-interior regions. These update mappings describe grain-level cavity redistribution governed by local diffusion and geometry.

The coarse-graining procedure, by contrast, is a spatial aggregation defined on an equal-time configuration. For a block, the effective cavity fraction is obtained by aggregating the instantaneous cavity fractions of the grain-scale constituents contained within that block. Provided that the cavity evolution mechanism remains unchanged across scales and that inter-block transport during a single update step is negligible, spatial aggregation does not alter the dynamical type of the evolution law. Instead, each higher-level block inherits an effective update rule induced by its composition. In this sense, Figure 3 provides the effective grain-scale dynamical kernel from which hierarchical evolution is constructed.

Here, the internal time step t characterizes the intrinsic relaxation time of cavity migration within a grain. In addition, the system involves a much longer characteristic time τ associated with the nucleation of new cavities. These two processes are assumed to satisfy

t≪τ.

Creep measurements are performed over an observational interval Δt, which can be chosen such that

t≪Δt≪τ.

Under this separation of time scales, cavity redistribution within each grain effectively completes within a single observational interval, while the generation of new cavities remains negligible at that scale. Consequently, at any macroscopic time slice, the cavity fraction of all grains can be regarded as synchronously defined and quasi-stationary with respect to the redistribution dynamics.

The hierarchical structure function is therefore constructed on equal-time spatial configurations. The coarse-graining operation acts purely as spatial aggregation, while the temporal evolution of the system is encoded in the slow variation in the fine-scale non-cavity volume fraction $\rho^{\left(0\right)}(t).$ Higher-level quantities are obtained through successive composition of Equation (6), which propagates the structural degradation across scales without reintroducing explicit fast migration dynamics.

Therefore, Figure 3 can be consistently regarded as providing the coarse-grained effective update rule that underlies the hierarchical equal-time structure function.

Using the presence or absence of grain boundaries within the hierarchical level corresponding regions as a criterion, different structure functions are divided. According to the description of creep microscopic mechanisms and behavior, cavity nucleation mostly occurs at grain boundaries, where the probability of nucleation at grain boundaries is significantly higher than that within grains. The mathematical representation of the structure function, for which the hierarchical level corresponding region contains at least one grain boundary, is expressed as a series system and belongs to Type I (since $\rho_{s}=1-\rho_{c}$, the curve in Figure 3a is transformed into Type I). If no grain boundaries are present in any of the lth level corresponding regions, the state of the (l+1)th level is considered failed when at least k states of the lth level fail. The mathematical representation of the structure function, which the hierarchical level corresponding region does not contain grain boundaries, is considered a k-out-of-n system and belongs to Type III.

**Figure 6:**
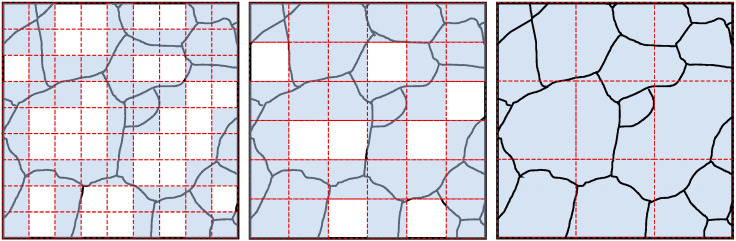
The schematic diagram for the determination of probability h(l/L). The white cavities and the blue cavities denote those within the grain and at boundaries, respectively.

As the creep hierarchical modeling involves multiple types of structure functions, it is necessary to consider the ratio of structure functions at different levels. Take two types (with Type I and Type III) of structure functions as an example for demonstration. In a system with L levels, the probability of structure function belonging to Type I at the lth level can be obtained by statistical methods, denoted as h(l/L), while the probability of the structure function belonging to Type III is 1−h(l/L). The probability h(l/L) depends on the sizes of the grains and the grain-boundary distribution in the material, which depict the disorders of the systems. It can be determined by observing the metallographic diagram of the material in practical engineering fields. Figure 6 briefly demonstrates the determination of probability h(l/L) in a single metallographic image. The higher hierarchical levels and corresponding increasing regions are arranged from left to right. The red dashed lines delineate the regions corresponding to different hierarchical levels, while the blue-filled areas represent the regions with grain boundaries. The structure functions of the blue-filled areas belong to Type I, and the ratio of the blue-filled area represents h(l/L). In fact, the internal microstructure of the same material is different, and the selection of metallographic diagrams will affect the determination of probability h(l/L). Therefore, multiple metallographic images or multiple regions in a metallographic image are usually selected for the above operations, and statistical methods are used to reduce the uncertainty.

### 2.3. Modeling with Different Structure Functions

In the creep hierarchical modeling method, the structure function has a significant impact on the modeling results. Next, further studies will be conducted on the creep rate curve modeling with only one type and multiple types of structure functions.

#### 2.3.1. Modeling with Only One Type of Structure Function

Modeling with only one type of structure function means that the structure functions are the same for all levels from microscale to macroscale. Based on Equation (7), assuming that the structure function $\phi^{\left(l\right)}=\phi$ for every level, a 2-out-of-3 system is selected for numerical demonstration, and the structure function is expressed as

(9) $$f_{\phi }\left(\rho \right)=3\rho^{2}-2\rho^{3}.$$

The microscopic survival probability is given as $\rho^{\left(0\right)}\left(t\right)=\exp\left[-{\left(t\right)}^{\alpha }\right]$, where α is a parameter that characterizes the variation in microscopic survival probability. The structure function of Type III generally shows a monotonically increasing creep rate curve [23]. Similar analyses are conducted for the structure functions of Type I and II, where the creep rate modeled by the Type-I structure function mostly gave a monotonically decreasing curve, while the creep rate modeled by the Type-II structure function showed a monotonically increasing trend. The related numerical results for the one-type model are presented in Appendix A. Based on the description of the creep microscopic mechanism, the Type-III structure function represents the evolution law of microscopic parameters during the creep damage process only within grains. This situation corresponds to single-crystal materials, which have a highly ordered internal microstructure without grain boundaries. Moreover, the monotonic increase in the creep rate curve of single-crystal materials has also been observed in actual experiments [53]. In fact, the creep rate curves observed in engineering are mostly bathtub-shaped, indicating that the effect of disorder plays an important role. Therefore, research on the modeling with multiple types of structure functions is required.

#### 2.3.2. Modeling with Multiple Types of Structure Functions

This section focuses on hierarchical modeling of the creep damage process with multiple types of structure functions, taking two types (with Type I and Type III) of structure functions as an example to investigate the creep rate curve. When multiple types of structure functions exist, this modeling method needs to consider the sequence s of the structure functions. When there are only two types of structure functions (denoted by ϕ and $\phi^{\prime }$), the system-level survival probability corresponding to the specific sequence s is expressed as

(10) $$\rho_{\left[s\right]}^{\left(L\right)}\left(\rho^{\left(0\right)}\right)=\underset{s_{n}}{\underbrace{f_{\phi }\circ f_{\phi }\circ \ldots f_{\phi }}}\circ f_{\phi^{\prime }}\circ \ldots \underset{s_{k}}{\underbrace{f_{\phi }\circ f_{\phi }\circ \ldots f_{\phi }}}\circ f_{\phi^{\prime }}\circ \ldots f_{\phi^{\prime }}\circ \underset{s_{0}}{\underbrace{f_{\phi }\circ f_{\phi }\circ \ldots f_{\phi }\left(\rho^{\left(0\right)}\right)}},$$

where the specific sequence $s=\left\{s_{n},s_{n-1},\ldots ,s_{0}\right\}$, which is described by the number of structure functions ϕ between the structure functions $\phi^{\prime }$. All possible sequences can be represented by different combinations of the given sequence s. If two adjacent structure functions $\phi^{\prime }$ exist, the corresponding value is denoted as 0. For ease of understanding, if the structure function composition is $\left(\phi ,\phi ,\phi^{\prime },\phi^{\prime },\phi \right)$, the sequence s={2,0,1}.

In a hierarchical system with L levels, the probability h(l/L) is used to characterize the possibility of structure function ϕ, while the possibility of the structure function $\phi^{\prime }$ is 1−h(l/L). The probability h(l/L) is used to calculate the probabilities ψ(s) of sequences. The macroscopic observable result is the weighted average of the survival probabilities with all possible sequences, which is expressed as

(11) $$\rho^{\left(L\right)}=\sum_{s}\psi \left(s\right)\rho_{\left[s\right]}^{\left(L\right)},$$

where the probability of sequence is calculated as

(12) $$\psi \left(s\right)=\prod_{j=0}^{n-1}\left[\left[1-h\left(\frac{j+\sum_{k=0}^{j}s_{k}}{L}\right)\right]\prod_{i_{j}=j+\sum_{k=0}^{j-1}s_{k}}^{j+\sum_{k=0}^{j-1}s_{k}-1}h\left(\frac{i_{j}}{L}\right)\right]\times \prod_{i=L-s_{n}+1}^{L}h\left(\frac{i}{L}\right).$$

The above expression appears complex, but its practical application is simple. For instance, a given sequence s is assumed as $\rho_{\left[\left\{1,2\right\}\right]}^{\left(L\right)}=f_{\phi }\circ f_{\phi^{\prime }}\circ f_{\phi }\circ f_{\phi }\left(\rho^{\left(0\right)}\right)$, and ψ({1,2})=h(4/4)[1−h(3/4)]h(2/4)h(1/4).

**Figure 7:**
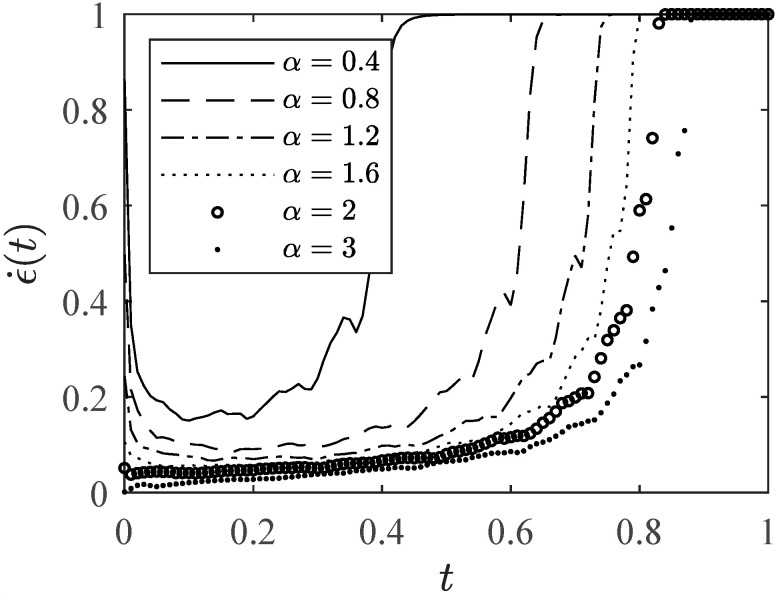
Creep rate curves obtained with different values of α.

Numerical experiments are conducted on the creep hierarchical modeling method with 2 types of structure functions. Given ϕ as the Type-I structure function with the probability h(l/L), the structure function in this case is $f_{\phi }\left(\rho \right)=\rho^{3}$. The Type-III structure function $\phi^{\prime }$ with the probability 1−h(l/L) is represented by $f_{\phi }(\rho )=3\rho^{2}-2\rho^{3}.$ The microscopic survival probability is set as $\rho^{\left(0\right)}\left(t\right)=\exp\left(-t^{\alpha }\right)$. The hierarchical level from microscale to macroscale is L=12, and the time interval is Δt=0.001. The probability of the structure function ϕ is represented by h(x)=1−(1−x)/2. A creep damage process from microscale to macroscale is modeled, and the normalized creep rate curve is presented in Figure 7.

Figure 7 illustrates that as parameter α increases, the leading edge of the bathtub-shaped curve gradually transitions from a steep drop to a more gradual slope, and the creep rate gradually becomes a monotonically increasing curve. Therefore, it is inferred that there exists a threshold condition for the shape of the creep rate curve.

### 2.4. Influence of Model Parameters

The creep hierarchical modeling method can reconstruct the creep damage process based on a parameter combination [α,k,n,L,Δt,h(l/L)]. Parameter α characterizes the variation in microscopic survival probability, parameters (k, n) represent the selection of the structure function, L is the number of hierarchical levels, Δt is the observation time interval, and h(l/L) characterizes the probability of the structure function at the lth level. Conducting a parameterized analysis on this modeling method can help to explore the impact of different parameters on the model results, providing guidance for investigating the theoretical threshold of the bathtub-shaped creep rate curve.

#### 2.4.1. The Rate of Microscopic Degradation

**Figure 8:**
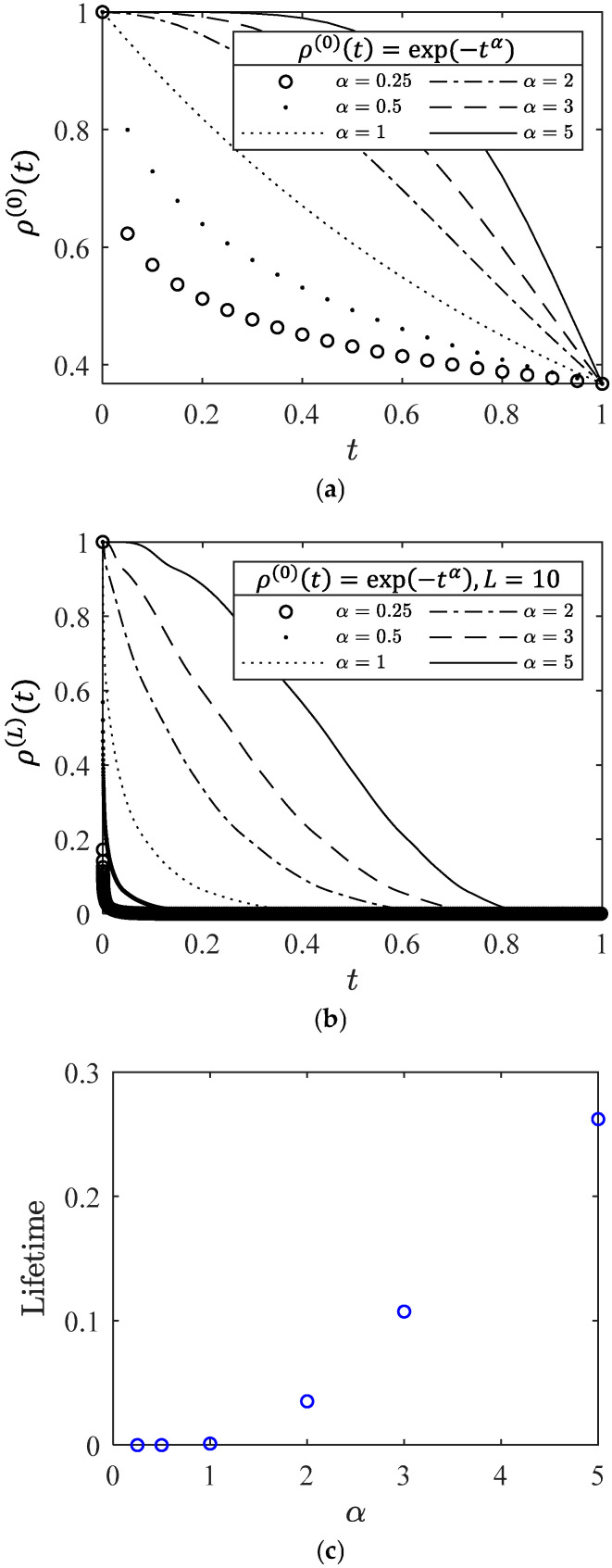

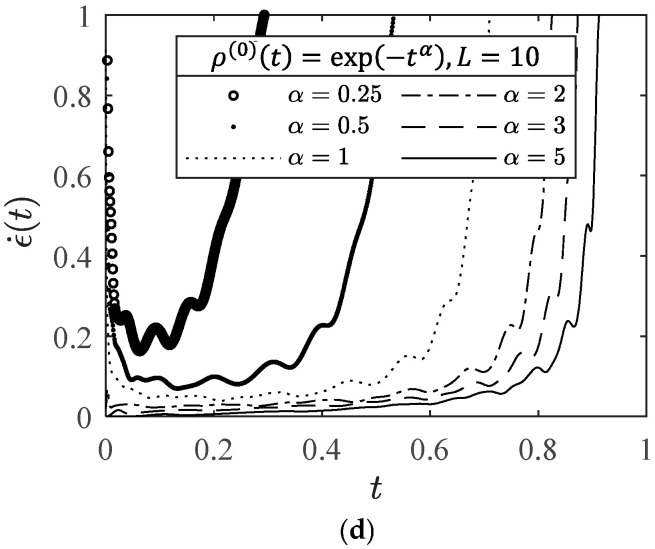
The influence of parameter α on (**a**) microscopic survival probability, (**b**) macroscopic survival probability with L=10, (**c**) lifetime, and (**d**) creep rate curve.

Assuming that the microscopic survival probability is described by $\rho^{\left(0\right)}\left(t\right)=\exp\left(-t^{\alpha }\right)$, where the parameter α serves as the unique description of the microscopic degradation process. It corresponds to the failure rate of the exponential distribution. Figure 8a illustrates the impact of parameter α on microscopic survival probability. Figure 8b demonstrates the impact of different parameter α values on macroscopic survival probability in a system with L=10. As the parameter α increases, the degradation of macroscopic survival probability $\rho^{\left(L\right)}(t)$ slows down. The survival probability of α=0.5 approaches zero within the same time frame, while the survival probability of α=5 begins to degrade from 1. Figure 8c shows that using a macroscopic survival probability of $\rho^{\left(L\right)}\left(t\right)\geq 0.5$ as a failure criterion provides the corresponding lifetime of the material, indicating that a larger α corresponds to a longer lifetime in a hierarchical system with L levels. The creep rate curves modeled by different values of parameter α are shown in Figure 8d. The curves for α=[0.25,0.5,1] exhibit a significant downward trend in the early stages of degradation, and gradually transition from bathtub-shaped to non-bathtub-shaped as parameter α increases. The parameter α appears to be closely linked to the theoretical threshold for the shape of creep rate curve.

#### 2.4.2. The Number of Hierarchical Levels

**Figure 9:**
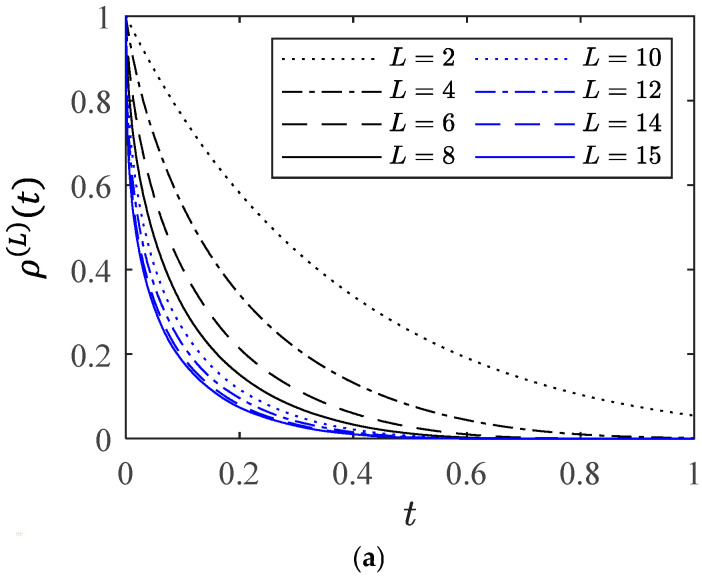

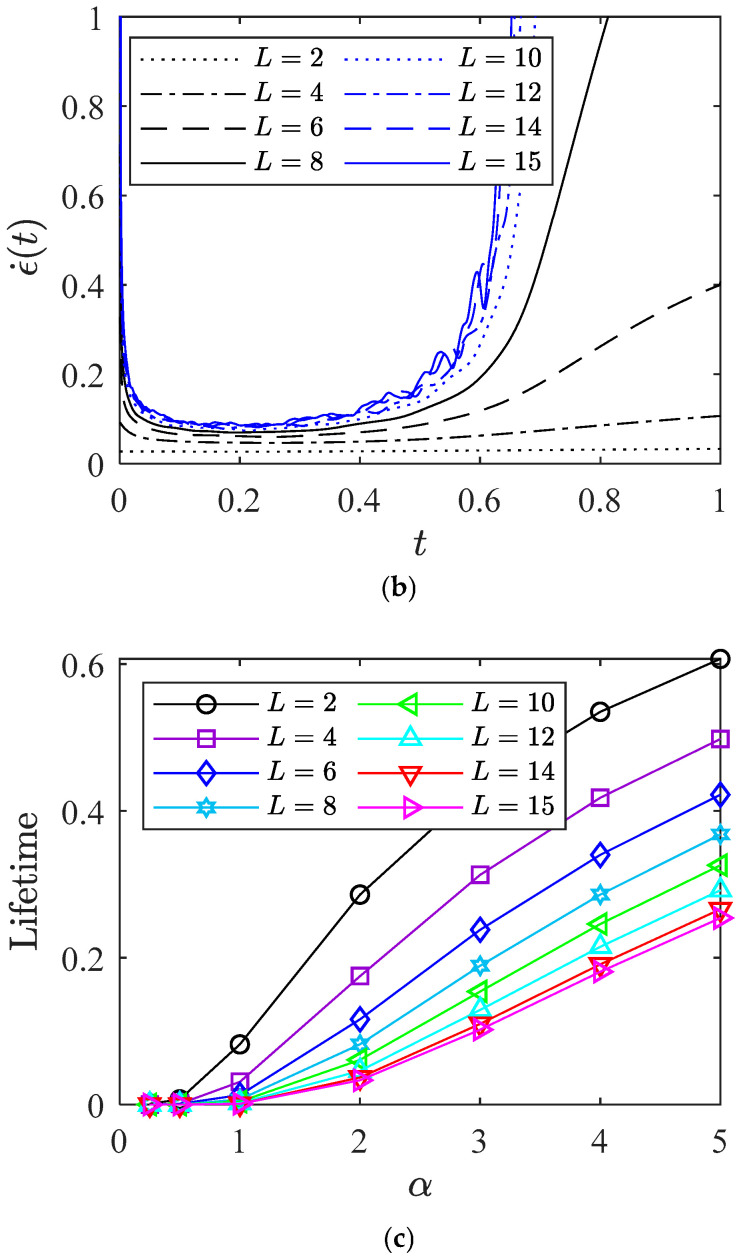
The influence of the hierarchical level number L on (**a**) macroscopic survival probability, (**b**) creep rate curve, and (**c**) lifetime.

The influence of the hierarchical level number L on macroscopic creep degradation behavior is investigated. Figure 9a,b demonstrate the influence of different L values on macroscopic survival probability and creep rate curve, respectively. Figure 9c shows the corresponding lifetimes. It can be seen that as L increases the results on the three quantities converge. Moreover, when L is large enough, the creep rate exhibits the so-called bathtub-shaped curve, and the higher the hierarchical level number L, the more pronounced the bathtub shape. The results shown in Figure 9 imply that the behavior of the system can be reflected using the microscopic parameters with proper structure functions and a sufficiently large number of levels.

#### 2.4.3. The Observation Time Interval

**Figure 10:**
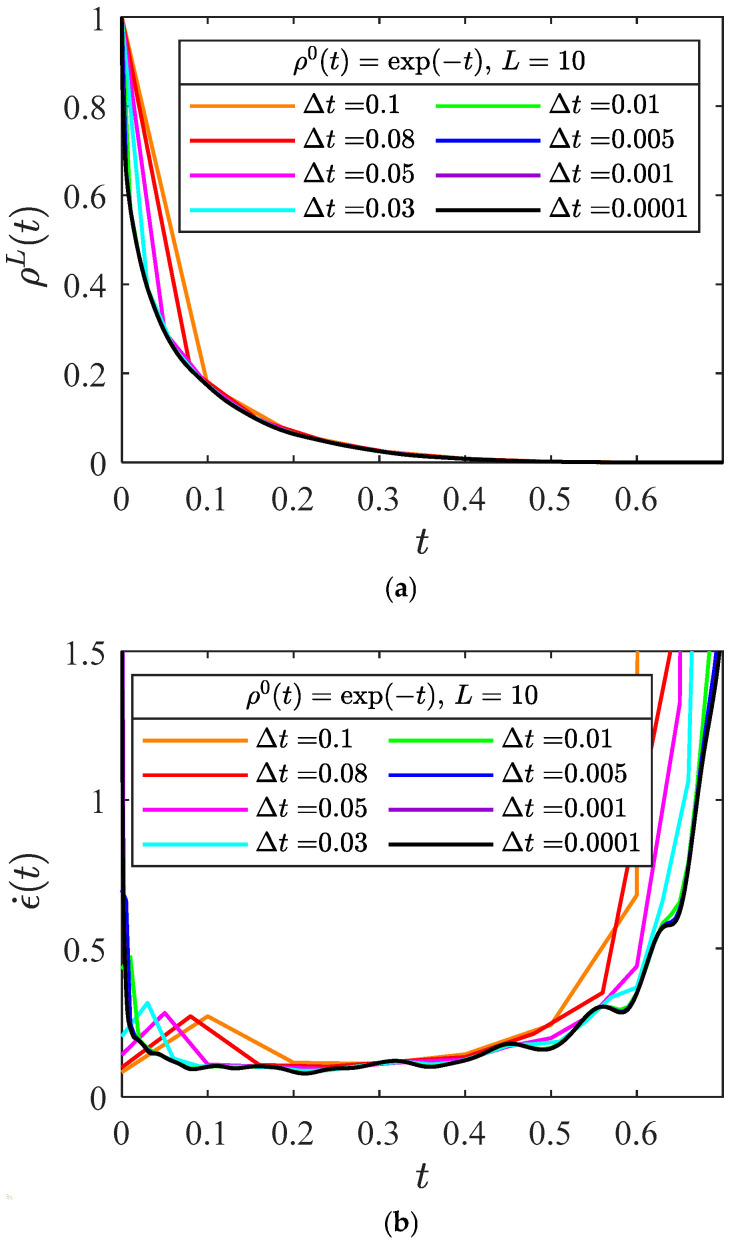
The influence of observation time interval Δt on (**a**) macroscopic survival probability and (**b**) creep rate curve.

The impact of observation time interval Δt is shown in Figure 10, and the observation time interval Δt mainly affects the early stage of creep damage process. A smaller observation time Δt represents richer information that is acquired for modeling. In the early stage of the creep damage process, the slope of $\rho^{L}(t)$ vs. t is steep, requiring a smaller time step to capture the variation in the curve. In the later stage, the slope is smaller, and a larger time step is sufficient to describe the curve. Nevertheless, as the whole process of creep is of interest, a proper time interval must be used such that the dynamical behavior of the curve can be fully and accurately captured.

## 3. Theoretical Threshold for the Emergence of the Bathtub-Shaped Creep Rate Curve

### 3.1. Early-Stage Asymptotic Behavior of the Creep Hierarchical Model

The theoretical threshold of the bathtub-shaped creep rate curve is further investigated based on the parametric study results in Section 2.4. By observing the modeled creep rate curves shown in Figure 7, Figure 8d and Figure 10b, it is evident that the creep rate curves exhibit expected behavior in the stage of tertiary creep. However, the creep rate curves in the stage of primary creep can be vastly different under different model parameters. For example, the curve associated with α=5 in Figure 8d fails to reproduce the sharp decrease in the creep strain rate in the stage of primary creep. Therefore, the theoretical threshold for producing the bathtub-shaped creep rate curve should be determined mainly by the early-stage behavior of the creep rate.

According to Equation (3), the creep rate can be rewritten based on the definition of survival probability ρ(t) as

(13) $$\dot{\epsilon }\left(t\right)\propto -\frac{dlnf_{\phi^{\left(L\right)}}\left(\rho^{\left(0\right)}\left(t\right)\right)}{d\rho^{\left(0\right)}\left(t\right)}\times \frac{d\rho^{\left(0\right)}}{dt}=-\frac{df_{\phi^{\left(L\right)}}\left(\rho^{\left(0\right)}\left(t\right)\right)}{d\rho^{\left(0\right)}\left(t\right)}\times \frac{1}{f_{\phi^{\left(L\right)}}\left(\rho^{\left(0\right)}\left(t\right)\right)}\times \frac{d\rho^{\left(0\right)}\left(t\right)}{dt}.$$

The time variable t in $\rho^{\left(0\right)}(t)$ is omitted as necessary without introducing ambiguity. In the stage of primary creep, the macroscopic and microscopic survival probabilities are both close to 1 and gradually decrease as creep damage accumulates. In this regime, $1/f_{\phi^{\left(L\right)}}(\rho^{\left(0\right)})$ varies slowly, while $-d\rho^{\left(0\right)}/dt$ also changes gradually and approaches a nearly constant value in the initial stage. Therefore, Equation (13) can be approximated in primary creep as

(14) $$\dot{\epsilon }\left(t\right)\approx \frac{df_{\phi^{\left(L\right)}}\left(\rho^{\left(0\right)}\right)}{d\rho^{\left(0\right)}}.$$

Using Equation (11), the creep rate function is the weighted average of all possible sequences and can be expressed as

(15) $$\dot{\epsilon }\left(t\right)\approx \sum_{s}\psi \left(s\right)\frac{d\rho_{\left[s\right]}^{\left(L\right)}}{d\rho^{\left(0\right)}}.$$

Therefore, the creep rate behavior in primary creep depends on the combined effects of the sequence probability ψ(s) and the derivative $d\rho_{\left[s\right]}^{\left(L\right)}/d\rho^{\left(0\right)}$.

**Figure 11:**
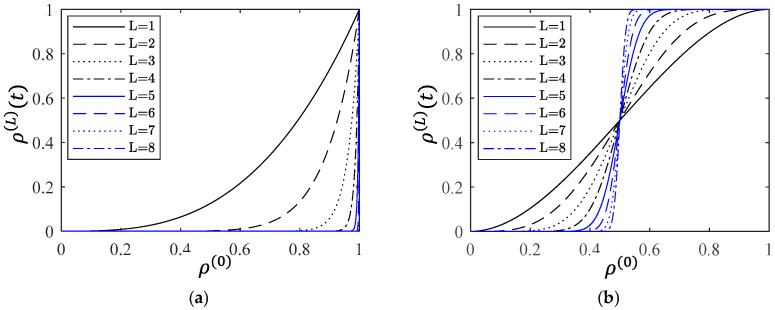
Creep damage behavior in terms of survival probability function $\rho^{\left(L\right)}(t)$ with different structure functions: (**a**) Type-I only. (**b**) Type-III only.

**Figure 12:**
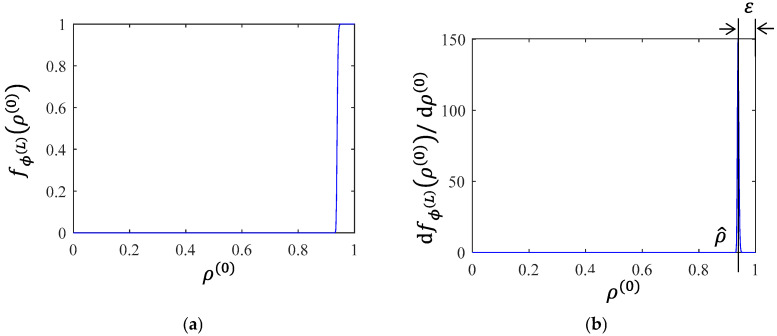
(**a**) The system-level survival probabilities of a specific sequence composed of the Type-I and Type-III structure functions and (**b**) the derivative of $f_{\phi^{\left(L\right)}}(\rho^{\left(0\right)})$ with respect to $\rho^{\left(0\right)}$ in (**a**).

Figure 11a,b illustrate the creep damage process in terms of survival probability function $\rho^{\left(L\right)}(t)$ with only Type-I or Type-III structure functions, respectively. It is worth noting that the microscopic survival probability $\rho^{\left(0\right)}(t)$ starts from 1 and gradually decreases to 0. Since the x-axis is microscopic survival probability $\rho^{\left(0\right)}$, the direction from right to left corresponds to the process of creep degradation. As $\rho^{\left(0\right)}(t)$ approaches 1, it can be seen that $df_{\phi^{\left(L\right)}}(\rho^{\left(0\right)})/d\rho^{\left(0\right)}$ approaches ∞ in the Type-I case and approaches 0 in the Type-III case. Figure 12a shows the creep damage process for a specific sequence s composed of Type-I and Type-III structure functions, and the corresponding derivative is shown in Figure 12b. In the stage of primary creep, $df_{\phi^{\left(L\right)}}(\rho^{\left(0\right)})/d\rho^{\left(0\right)}$ has a peak value at $\hat{\rho }$, and there exists a parameter $\varepsilon =1-\hat{\rho }$ where 0<ε≪1.

**Figure 13:**
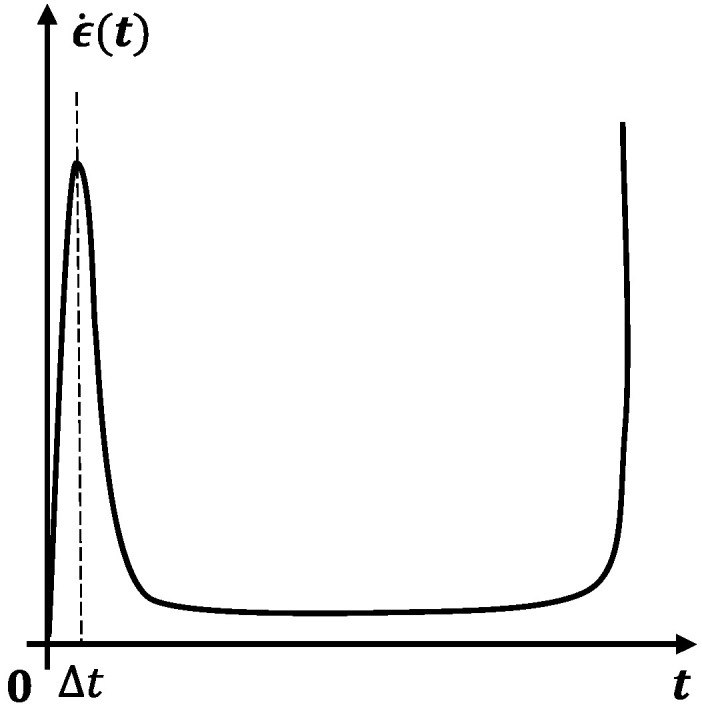
Illustration of the theoretical creep rate curve.

For the sequence containing both Type-I and Type-III structure functions, the peak position satisfies $\hat{\rho }\in (0.5,1)$. This means that when ρ=1, the creep process corresponding to any sequence has not yet started. Therefore, at t=0, $f_{\phi^{\left(L\right)}}(\rho^{\left(0\right)})=1$ and $df_{\phi^{\left(L\right)}}(\rho^{\left(0\right)})/d\rho^{\left(0\right)}=0$, which implies $\dot{\epsilon }\left(0\right)=0$. As the creep degradation of some sequences s begins, the modeled creep damage behavior depends on the weighted average of the changes associated with each sequence $s_{i}$. Consequently, the theoretical creep rate curve resulting from the creep hierarchical modeling method resembles the curve illustrated in Figure 13. This theoretical curve represents the intrinsic characteristic of the creep rate curve determined by the creep hierarchical modeling method utilizing at least two types of microscopic creep damage mechanisms. Notably, the actual creep rate data on CMSX-4 alloy at 750 °C and 750 MPa reported in Ref. [53] exhibits a similar curve shown in Figure 13.

By comparing the theoretical curve in Figure 13 with the classical bathtub-shaped creep rate curve observed in practice, it is seen that the difference mainly depends on the first observation point Δt and the initial creep rate value $\dot{\epsilon }\left(\Delta t\right)$. Therefore, the theoretical threshold for the observed bathtub-shaped creep rate curve can be investigated through these two quantities. For clarity, only the key asymptotic results are retained in the main text, while the detailed sequence-based derivation is provided in Appendix B.

Using the asymptotic analysis in Appendix B, the structure function near $\rho^{\left(0\right)}=1$ can be approximated by $f_{\phi }\left(1-\varepsilon \right)\approx 1-a\varepsilon$ for the Type-I structure function, and $f_{\phi^{\prime }}\left(1-\varepsilon \right)=1-b\varepsilon^{c}$ for the Type-III structure function, where a, b, and c are positive integers. Substituting these approximations into Equation (7) and performing the calculation yields the minimum strain $\varepsilon_{\min}\approx a^{-L}.$ This expression represents the minimum effective degradation scale generated by the hierarchical structure in the primary creep stage, and it provides the basis for determining whether the initial decrease in the creep rate can be captured in actual observations.

### 3.2. Threshold Conditions for the Observed Bathtub-Shaped Creep Rate Curve

The observed bathtub-shaped creep rate curve depends on whether the intrinsic early-stage transition in Figure 13 can be captured in practice. From the perspective of the creep hierarchical modeling method, the corresponding threshold conditions are determined by the first observation time interval Δt and the initial creep rate function $\dot{\epsilon }\left(\Delta t\right)$.

#### 3.2.1. The Minimal Observation Time Interval Δt

To satisfy the initial creep rate function $\dot{\epsilon }\left(\Delta t\right)\gg 0$, the first observation time t=Δt should be sufficiently larger than the minimum effective degradation scale. Based on the previous discussion, there exists at least one peak position $\hat{\rho }$ of sequence s at the first observation time t=Δt, which means $\Delta t\gg t_{\varepsilon_{\min}}$. Since survival probability $\rho^{\left(L\right)}(t)$ typically changes slower than t in practical primary creep, the condition is transformed into $\Delta t\gg \varepsilon_{\min}$. Substituting $\varepsilon_{\min}\approx a^{-L}$ gives

(16) $$-\frac{\log_{a}\;\Delta t}{L}\ll 1.$$

This condition indicates that the first observation interval should be sufficiently large compared with the minimum degradation scale generated by the hierarchical structure. Otherwise, the early rapid increase in creep rate is still captured, and the curve is no longer bathtub-shaped.

#### 3.2.2. The Initial Creep Rate Function $\dot{\epsilon }\left(\Delta t\right)$

The initial creep rate function $\dot{\epsilon }\left(\Delta t\right)\gg 0$ is a necessary condition for observing the classical bathtub-shaped curve. Using Equations (3) and (11), the creep rate function at t=Δt can be expressed as

(17) $$\begin{array}{ll} \dot{\epsilon }\left(\Delta t\right) & \propto -\frac{1}{\rho^{\left(L\right)}\left(0\right)}\frac{\rho^{\left(L\right)}\left(\Delta t\right)-\rho^{\left(L\right)}\left(0\right)}{\Delta t}\\ & =\frac{1}{\Delta t}\int_{\rho^{\left(0\right)}\left(\Delta t\right)}^{1}\frac{d\rho^{\left(L\right)}}{d\rho^{\left(0\right)}}d\rho^{\left(0\right)}=\frac{1}{\Delta t}\int_{\rho^{\left(0\right)}\left(\Delta t\right)}^{1}\left(\sum_{s}\psi \left(s\right)\frac{d\rho_{\left[s\right]}^{\left(L\right)}\left(\rho^{\left(0\right)}\right)}{d\rho^{\left(0\right)}}\right)d\rho^{\left(0\right)} \end{array}.$$

Therefore, separate asymptotic approximations are needed for $\sum_{s}\psi \left(s\right)$ and $\frac{d\rho_{\left[s\right]}^{\left(L\right)}(\rho^{\left(0\right)})}{d\rho^{\left(0\right)}}$. Using the asymptotic derivation in Appendix B, the derivative term satisfies

(18) $$\frac{d\rho_{\left[s\right]}^{\left(L\right)}}{d\rho^{\left(0\right)}}{|}_{\rho^{\left(0\right)}={\hat{\rho }}^{\left(0\right)}}\propto \frac{1}{\varepsilon },$$

while the sequence probability term satisfies

(19) $$\sum_{s}\psi \left(s\right)\approx \varepsilon^{-\log_{a}\;h\left(0\right)}.$$

Substituting these approximations into Equation (17) yields

(20) $$\dot{\epsilon }\left(\Delta t\right)\approx \frac{1}{\Delta t}\int_{0}^{{\left(\Delta t\right)}^{\alpha }}\varepsilon^{-\log_{a}\;h\left(0\right)-1}d\varepsilon \approx \frac{1}{\Delta t}{\left(\Delta t\right)}^{-\alpha \;\log_{a}\;h\left(0\right)}∼{\Delta t}^{-\alpha \;\log_{a}\;h\left(0\right)-1}.$$

For a classical bathtub-shaped curve, the creep rate curve should decrease rapidly as Δt approaches 0. In this case, it is required that [40]

(21) $$-\alpha \;\log_{a}\;h\left(0\right)-1<0.$$

In summary, a classical bathtub-shaped creep rate curve is presented when the conditions of Equations (16) and (21) are satisfied. Observing the theoretical thresholds, it can be seen that the shape of the creep rate curve is the result of the combined effects of multiple microscopic parameters. This study reveals the statistical physics of the phenomenological creep rate curve by the creep hierarchical modeling method and provides a new research perspective for the shape of the creep rate curve.

## 4. Method Validation with Experimental Data

In this section, the parameter combinations in the proposed creep hierarchical modeling method are inferred from experimental creep damage data to demonstrate the capability of the method for bottom-up modeling of creep degradation processes. To examine the applicability of the framework to different material systems, validations were performed using both metallic-material data and polymer-bonded composite data reported in the literature.

### 4.1. Validation Using Metallic-Material Creep Data

**Figure 14:**
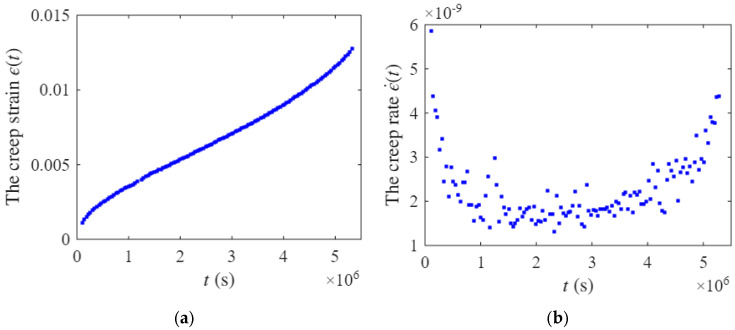
The experimental data of (**a**) creep strain and (**b**) creep rate in Ref. [54].

Ref. [54] reported creep data for Co-28Cr-6Mo-0.23C-0.17N alloy rods fabricated by electron beam melting (EBM), with the cylinder axis oriented along the build direction. In the present study, the creep strain-time data at 240 MPa and 650 °C from Ref. [54] were selected as a representative metallic-material case for model validation. The corresponding experimental creep response is shown in Figure 14a. Using the experimental strain-time data, the creep rate is calculated by the forward finite difference scheme as

(22) $$\dot{\epsilon }\left(t_{i}\right)=\left(\epsilon_{i+1}-\epsilon_{i}\right)/\left(t_{i+1}-t_{i}\right),$$

where $\epsilon_{i}$ and $t_{i}$ are the ith testing data point. The resulting creep rate curve is shown in Figure 14b.

Given the parameter combination [α,k,n,L,Δt,h(l/L)], the creep degradation behavior was described by the proposed hierarchical modeling method. Based on Equation (3), the survival probability is proportional to the creep rate. Therefore, both the experimental data and the modeling results are normalized. Specifically, the highest creep rate value in the primary creep stage and the minimum value of the measured creep rate data were used as the normalization reference for both the experimental and modeling results. The parameter identification is conducted by minimizing the error sum of squares through nonlinear optimization, with the stopping criterion set such that the size of the current step is less than ${1\times 10}^{-6}$.

During parameter inference, the full parameter combination leads to relatively high computational cost. Since h(l/L) is a functional quantity and cannot be directly optimized by the nonlinear optimization procedure, it is specified as

(23) h(l/L)=1−(1−l/L)/2.

**Figure 15:**
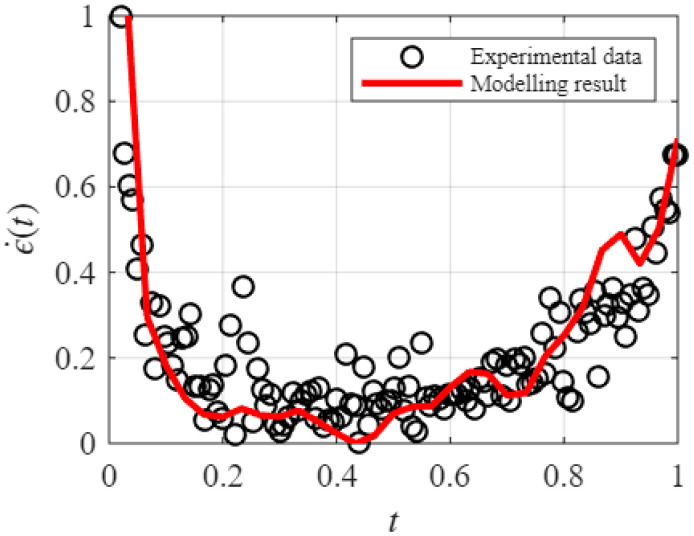
The experimental data and modeling result of creep rate curve.

The parameter Δt, representing the monitoring time interval in the degradation process, is set to Δt=0.01. By optimizing the parameter combination (k,n,L,α), a local optimal solution is obtained for the metallic-material dataset, with an error sum of squares of 1.3088. This solution corresponds to the parameter set (α=1.3566,k=2,n=4,L=12) and the resulting comparison between the experimental data and the modeling results is presented in Figure 15.

It should be noted that the original numerical dataset was not available in Ref. [54]. Therefore, the experimental data used for validation in this section were digitized from the published figure. As a result, the fitted curve exhibits a certain degree of fluctuation, which may be attributed to two factors. First, the fitting performance is influenced by the inherent scatter of the experimental data itself. Second, minor numerical deviations may have been introduced during the data digitization process. Nevertheless, the fitted curve successfully captures the characteristic three-stage creep evolution of the metallic material, including the primary, secondary, and tertiary stages. This suggests that the proposed creep hierarchical modeling method is applicable to full-process creep modeling of metallic materials. In future work, systematic benchmark comparisons with conventional methods will be carried out using directly measured experimental datasets.

### 4.2. Validation Using Polymer-Bonded Composite Creep Data

**Figure 16:**
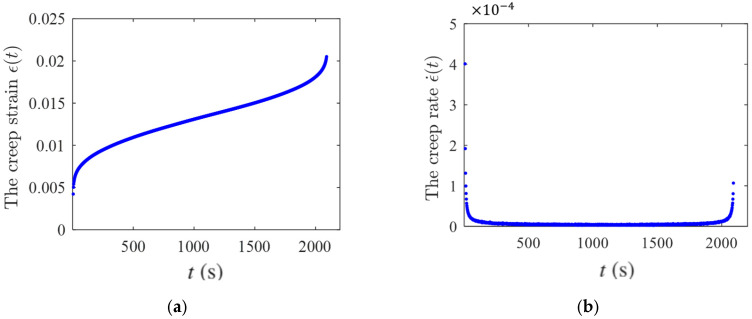
The experimental data of (**a**) creep strain and (**b**) creep rate in Ref. [7].

Duan [7] reported uniaxial compression creep data for polymer-bonded composite materials consisting of 94 wt.% barium sulfate filler and 6 wt.% Fluororubber matrix. The creep strain-time data at 40 °C and 17.5 MPa from Ref. [7] were used here as a representative case for model validation, as shown in Figure 16a. These data were extracted from the literature rather than obtained in the present work. Although Ref. [7] presented creep data for six specimens, no statistical information, such as repeated-test results, error bars, or dispersion measures, was reported for the selected condition. Hence, the present validation is based on the representative experimental curve available in the literature, and the absence of quantified statistical variation is a limitation of the current comparison. The creep rate was obtained from the experimental strain-time data using the same difference method as described in Section 4.1 and the resulting $\dot{\epsilon }(t)$ vs. t curve is shown in Figure 16b. The same normalization procedure, optimization strategy, and functional form of h(l/L) as those used in Section 4.1 were adopted here. For this composite-material case, the parameter Δt was set to $\Delta t=5\times 10^{-5}.$

**Figure 17:**
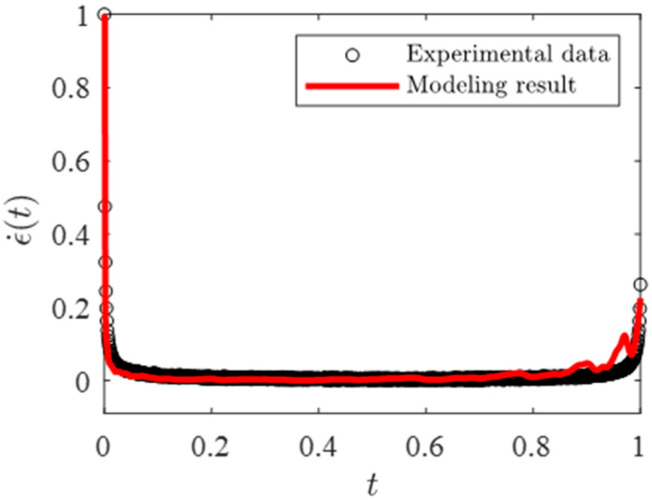
The experimental data and modeling result of creep rate curve.

By optimizing the parameter combination (k,n,L,α), a local optimal solution with an error value of 0.5369 was obtained. This corresponds to the parameter combination of α=0.84831, (k,n)=(2,6), and L = 13, as shown in Figure 17 for comparison. Based on the comparison between the experimental data and the modeling results, it can be observed that the proposed model provides a fundamental description of the three-stage degradation process during creep. The predicted responses in the primary and secondary creep stages are relatively stable and remain generally consistent with the experimental data. In the tertiary creep stage, the modeling results exhibit a fluctuation-like increase, whereas the experimental data show a smoother and more rapid increase; nevertheless, the overall accelerating trend is captured consistently by the model. It should be noted that, in the present framework, the initial basic elements are assumed to have identical failure rates. Therefore, the deviation appearing in the late stage is not attributed to an initially non-uniform distribution of failure properties at the most elementary level. A possible explanation is that, during the hierarchical aggregation and failure-transmission process, some intermediate-level effective components may evolve to exhibit relatively higher effective failure rates. As creep damage accumulates and the system approaches global failure, the influence of these intermediate-level weak components can be amplified through the hierarchical k/n structural interaction, leading to a non-smooth or fluctuation-like increase in the predicted creep rate in the tail region. In addition, the tertiary creep regime is intrinsically sensitive to experimental scatter, local microstructural heterogeneity, and stochastic damage coalescence near rupture, which are only represented in an effective coarse-grained manner in the present model. Therefore, although some discrepancy remains in the final stage, the proposed creep hierarchical modeling method is still capable of reproducing the overall shape of the creep rate curve and reflecting the essential creep degradation behavior of materials.

## 5. Conclusions

This study develops a hierarchical modeling framework for the full-stage creep damage process from microscale to macroscale based on statistical physics. Unlike conventional phenomenological or segmented approaches, the proposed method provides a unified and physically interpretable formulation that links microscopic creep failure mechanisms with macroscopic creep rate evolution. The model is further validated using representative metallic and composite creep data, with residual sums of squares of 1.3088 and 0.5369, respectively, showing its capability to reasonably reproduce the overall three-stage creep rate evolution. The main conclusions are summarized as follows. Based on the current research results, the following conclusions are drawn.

(1) A mechanism-based modeling method for the full-stage creep damage process is developed. This method provides a new alternative to conventional phenomenological and segmented creep models.
(2) A hierarchical connection between microscopic creep failure mechanisms and macroscopic creep damage behavior is established based on statistical physics, providing a physically interpretable framework for understanding the micro-mechanisms and interactions underlying creep degradation in structural materials.
(3) A theoretical threshold for the bathtub-shaped creep rate curve is derived through hierarchical modeling parameters. The corresponding conditions, $-(\log_{a}\;\Delta t)/L\ll 1$ and $-\alpha \;\log_{a}\;h\left(0\right)-1<0$, provide a new theoretical basis for interpreting the emergence of the creep rate curve shape from a microscopic perspective.

Despite these advantages, the proposed framework remains a coarse-grained and bottom-up model. Its accuracy mainly depends on the mathematical description of the underlying creep micro-damage mechanisms, and it does not explicitly resolve all detailed microstructural processes involved in creep damage. In addition, some model parameters still require calibration from experimental data, and the applicability of the method to more complex loading conditions, multiaxial stress states, and localized failure processes requires further investigation. It should also be noted that the present work is not intended as a comprehensive benchmarking study against classical phenomenological creep laws, such as the Norton law, or other conventional formulations. Rather, its main contribution is to provide a unified and physically interpretable framework for full-stage creep rate modeling by linking microscopic cavity evolution to macroscopic creep behavior. Within this framework, the characteristic features of the primary, secondary, and tertiary creep stages can be reproduced naturally as emergent outcomes of hierarchical damage evolution, rather than being imposed through separate constitutive forms for different stages.

Future work will focus on improving the representation of creep micro-mechanisms based on relevant experimental and theoretical studies, especially for materials with complex microstructures or multi-phase constituents. In particular, the present framework can be extended by incorporating phase-dependent microscopic evolution laws and heterogeneous hierarchical structures to better describe constituent interactions, interface effects, and localized damage processes. Systematic quantitative comparisons with conventional creep models under the same datasets and loading conditions will also be carried out. Moreover, the applicability of the present framework to long-term creep life prediction in engineering components will be investigated by coupling it with structural analysis methods and validating its predictive capability against long-term creep data under service-relevant loading and thermal conditions. The framework will also be generalized to other damage mechanisms, such as fatigue–creep interaction, oxidation-assisted creep damage, grain-boundary embrittlement, and other environmentally or microstructurally coupled degradation processes.

## Figures and Tables

**Figure A1:**
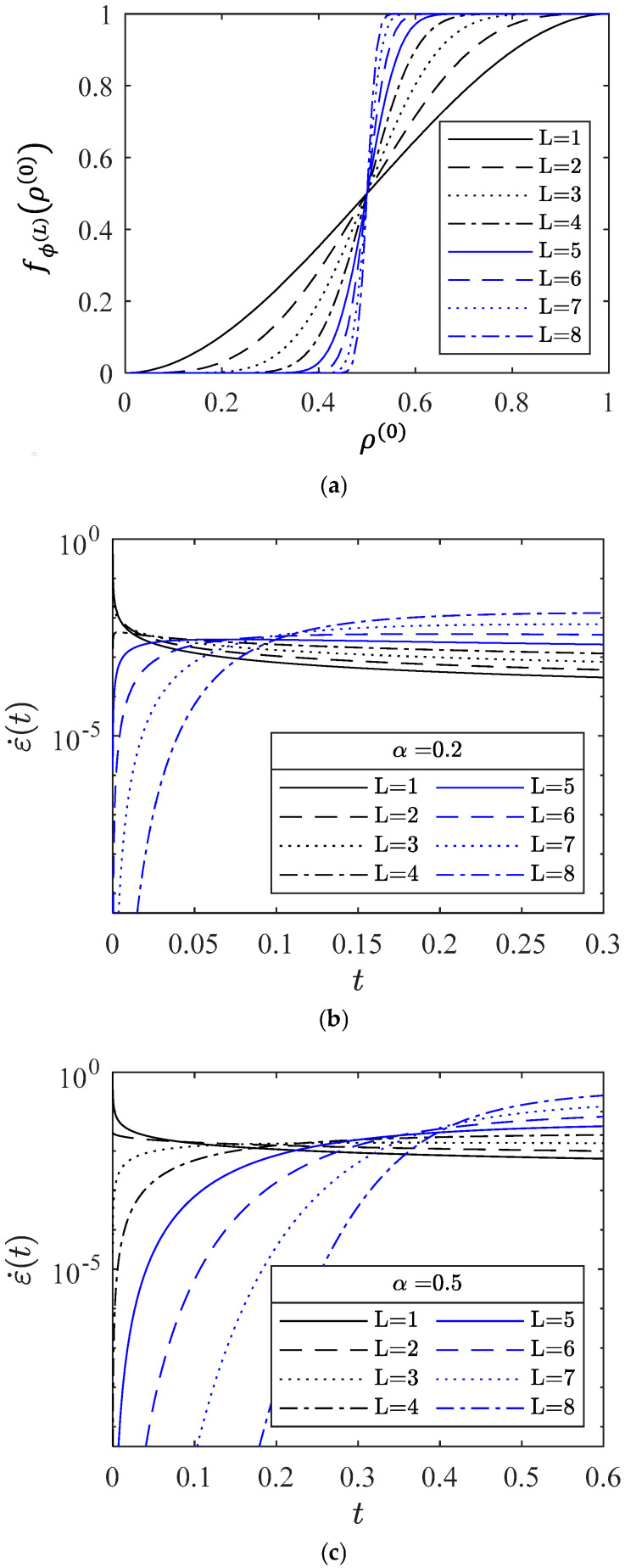
Modeling of creep damage process and creep rate function with only one type of structure function: (**a**) survival probabilities and creep rate with (**b**) α=0.2 and (**c**) α=0.5.

**Figure A2:**
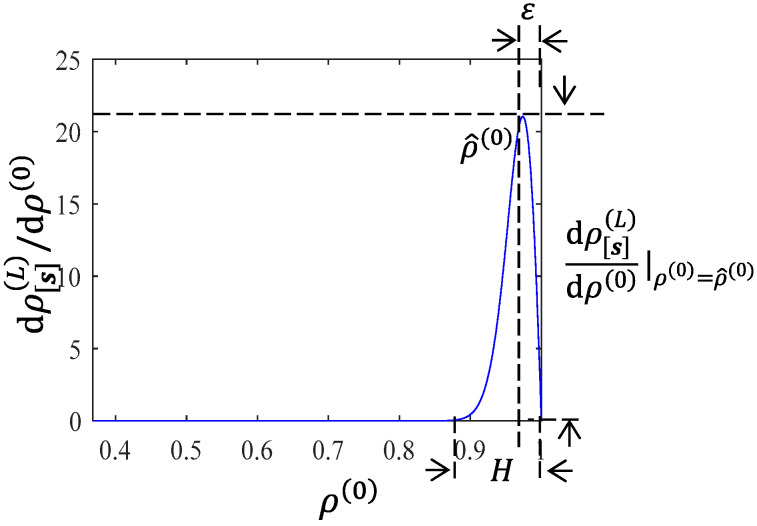
Schematic diagram of the parameters.

## Data Availability

The original contributions presented in this study are included in the article. Further inquiries can be directed to the corresponding author.

## Nomenclature

$\rho_{c}(t)$	The ratio of cavities per unit length, area, or volume.
$\rho^{\left(l\right)}(t)$	The survival probability function at the lth level.
t	A time increment variable.
$\phi^{\left(l\right)}$	Structure function at the lth level.
L	Number of hierarchical levels from microscale to macroscale.
$f_{\phi^{\left(l\right)}}\left(\cdot \right)$	Function of the survival probability of lth level as a function of all components at the (l−1)th level.
s	Sequence of structure function from microscale to macroscale.
$\epsilon (t),\dot{\epsilon }(t)$	Creep strain and creep rate, where $\epsilon (t)\propto -\frac{\Delta \rho^{\left(L\right)}\left(t\right)}{\rho^{\left(L\right)}\left(t\right)}.$
h(l/L)	Probability of structure function belonging to Type I at the lth level.
ψ(s)	Probability of sequence s.
∘	Operator that is represented as f∘g=f(g(t)).

## Appendix A

The microscopic survival probability is given as $\rho^{\left(0\right)}\left(t\right)=\exp\left[-{\left(t\right)}^{\alpha }\right]$, where α is a parameter that characterizes the variation in microscopic survival probability. For demonstration purposes, α is set as 0.5 or 0.2, L=8, and the time interval Δt=0.001. A bottom-up creep damage process modeling is conducted, and the survival probabilities and creep rate curves of each level are given in Figure A1. Based on Equation (3), it can be concluded that the creep rate is proportional to the system-level survival probabilities. Therefore, normalizing the survival probabilities is reasonable to be used for the observation of the creep rate curve shape.

Figure A1a demonstrates that for L≫1, the macroscopic survival probability rapidly decreases, a phenomenon known as “percolation” (if L becomes infinite), which becomes more pronounced as L increases. By comparing Figure A1b,c, it can be observed that both parameters α and L have an impact on the shape of the creep rate curve, and there are significant differences in the creep rate curves as α varies, particularly for L=2,3 and 4. This suggests that with an increasing L, the sensitivity of the creep rate curve shape to the microscopic degradation parameters decreases.

## Appendix B

This appendix derives the theoretical threshold for the observability of a bathtub-shaped creep rate curve, based on a set of key approximations.

### Appendix B.1. Approximation of the Hierarchy-Generated Scale ϵ

Let $\rho^{\left(0\right)}=1-\epsilon ,\;0<\epsilon \ll 1$. The structure function $f_{\phi }\left(1-\varepsilon \right)$ is represented by the k-out-of-n system and can be approximated by neglecting the higher-order terms of ε in the Taylor expansion. The Type-I structure function ϕ can be expressed as $f_{\phi }\left(1-\varepsilon \right)\approx 1-a\varepsilon$. For example, a series system is represented as $f_{\phi }\left(1-\varepsilon \right)=C_{3}^{3}\varepsilon^{3-3}{\left(1-\varepsilon \right)}^{3}=1-3\varepsilon +3\varepsilon^{2}-\varepsilon^{3}\approx 1-3\varepsilon$. The Type-III structure function $\phi^{\prime }$ can be expressed as $f_{\phi^{\prime }}\left(1-\varepsilon \right)=1-b\varepsilon^{c}$. For example, a 2-out-of-3 system is denoted as $f_{\phi^{\prime }}\left(1-\varepsilon \right)=C_{3}^{3}\varepsilon^{3-3}{\left(1-\varepsilon \right)}^{3}+C_{3}^{2}\varepsilon^{3-2}{\left(1-\varepsilon \right)}^{2}=1-3\varepsilon^{2}+2\varepsilon^{3}\approx 1-3\varepsilon^{2}$. The coefficients a,b and c are all positive integers.

The structure function ϕ for the first $s_{0}$ levels in the sequence $s=\left\{s_{n},s_{n-1},\ldots ,s_{0}\right\}$ is expressed as

(A1) $$\begin{array}{ccc} Layer\;l=0 & \rho^{\left(0\right)} & =1-\varepsilon \\ Layer\;l=1 & f_{\phi^{\left(1\right)}} & \approx 1-a\varepsilon \\ Layer\;l=2 & f_{\phi^{\left(2\right)}}\circ f_{\phi^{\left(1\right)}}=1-a\left[1-\left(1-a\varepsilon \right)\right] & \approx 1-a^{2}\varepsilon \\ \vdots & \vdots & \\ Layer\;l=s_{0} & f_{\phi^{\left(s_{0}\right)}}\circ f_{\phi^{\left(s_{0}-1\right)}}\circ \ldots \circ f_{\phi^{\left(1\right)}} & \approx 1-a^{s_{0}}\varepsilon . \end{array}$$

The structure function in the ($s_{0}+1$)th level is $\phi^{\prime }$, and the creep hierarchical modeling is further expressed as

(A2) $$f_{{\phi^{\prime }}^{\left(s_{0}+1\right)}}\circ f_{\phi^{\left(s_{0}\right)}}\circ f_{\phi^{\left(s_{0}-1\right)}}\circ \ldots \circ f_{\phi^{\left(1\right)}}=1-b{\left(a^{s_{0}}\varepsilon \right)}^{c}\approx 1-b\times a^{s_{0}c}\varepsilon^{c}.$$

The survival probability according to the sequence $\left\{{s_{1},s}_{0}\right\}$ is represented as

(A3) $$f_{\phi^{\left(s_{0}+1+s_{1}\right)}}\circ f_{\phi^{\left(s_{0}+s_{1}\right)}}\ldots \circ f_{\phi^{\left(s_{0}+2\right)}}\circ f_{{\phi^{\prime }}^{\left(s_{0}+1\right)}}\circ f_{\phi^{\left(s_{0}\right)}}\ldots \circ f_{\phi^{\left(1\right)}}\approx 1-a^{s_{1}}ba^{s_{0}c}\varepsilon^{c}.$$

The hierarchical modeling according to the sequence $\left\{0,{s_{1},s}_{0}\right\}$ is represented as

(A4) $$\begin{array}{l} f_{{\phi^{\prime }}^{\left(s_{0}+1+s_{1}+1\right)}}\circ f_{\phi^{\left(s_{0}+1+s_{1}\right)}}\circ f_{\phi^{\left(s_{0}+s_{1}\right)}}\ldots \circ f_{\phi^{\left(s_{0}+2\right)}}\circ f_{{\phi^{\prime }}^{\left(s_{0}+1\right)}}\circ f_{\phi^{\left(s_{0}\right)}}\ldots \circ f_{\phi^{\left(1\right)}}\\ \approx 1-b\times {\left(a^{s_{1}}ba^{s_{0}c}\varepsilon^{c}\right)}^{c}=1-b^{2}a^{s_{1}c+s_{0}c^{2}}\varepsilon^{c^{2}} \end{array}.$$

Similarly, the survival probability $\rho^{\left(L\right)}(t)$ modeled according to the sequence $\left\{s_{n},s_{n-1},\ldots ,s_{0}\right\}$ can be expressed as

(A5) $$\begin{array}{ll} \rho_{\left[s\right]}^{\left(L\right)} & \approx f_{\phi^{\left(\sum_{j=0}^{n}s_{j}+n\right)}}\circ f_{\phi^{\left(\sum_{j=0}^{n}s_{j}+n-1\right)}}\ldots \circ f_{\phi^{\left(s_{0}+2\right)}}\circ f_{{\phi^{\prime }}^{\left(s_{0}+1\right)}}\circ f_{\phi^{\left(s_{0}\right)}}\ldots \circ f_{\phi^{\left(1\right)}}\\ & \approx 1-b^{n}a^{s_{n-1}c+\ldots +s_{0}c^{n}}\varepsilon^{c^{n}}=1-b^{n}a^{\sum_{j=0}^{n-1}s_{j}c^{n-j}\varepsilon^{c^{n}}} \end{array},$$

where ${\hat{\rho }}_{\left[s\right]}^{\left(L\right)}=1-\varepsilon$ exists in creep damage process. Therefore, $\varepsilon \approx b^{n}a^{\sum_{j=0}^{n-1}s_{j}c^{n-j}}\varepsilon^{c^{n}}$ and after simplification, $\varepsilon \approx {\left(b^{n}\right)}^{\frac{1}{-c^{n}}}a^{-\sum_{j=0}^{n-1}s_{j}c^{-j}}$. Since the coefficients b,c, and n are all positive integers, ${\left(b^{n}\right)}^{\frac{1}{-c^{n}}}$ is a constant coefficient. Therefore, there exists $\varepsilon \propto a^{-\sum_{j=0}^{n-1}s_{j}c^{-j}}$. The maximum value of $\sum_{j=0}^{n-1}s_{j}c^{-j}=s_{0}+s_{1}c^{-1}+s_{2}c^{-2}+\cdots$ corresponds to $\varepsilon_{\min}$. When only the structure function ϕ (i.e., $s_{0}=L$) is present, ε obtains the minimum value of $\varepsilon_{\min}\approx a^{-L}$. Therefore, the approximate expression of parameter ε can be given as

(A6) $$\varepsilon \approx a^{-s_{0}}.$$

### Appendix B.2. Approximation of $\frac{d\rho_{\left[s\right]}^{\left(L\right)}(\rho^{\left(0\right)})}{d\rho^{\left(0\right)}}$

Figure A2 provides a schematic diagram of the relevant parameters. It is known that

(A7) $$\int_{\rho^{\left(0\right)}=0}^{1}\frac{d\rho_{\left[s\right]}^{\left(L\right)}\left(\rho^{\left(0\right)}\right)}{d\rho^{\left(0\right)}}d\rho^{\left(0\right)}=\rho_{\left[s\right]}^{\left(L\right)}\left(1\right)-\rho_{\left[s\right]}^{\left(L\right)}\left(0\right)=1,$$

which implies that the area

(A8) $$A_{\frac{d\rho_{\left[s\right]}^{\left(L\right)}}{d\rho^{\left(0\right)}}}=1\approx \frac{1}{2}\frac{d\rho_{\left[s\right]}^{\left(L\right)}}{d\rho^{\left(0\right)}}{|}_{\rho^{\left(0\right)}={\hat{\rho }}^{\left(0\right)}}\times H.$$

Due to ε≪1 and H∝ε, it follows that

(A9) $$\frac{d\rho_{\left[s\right]}^{\left(L\right)}}{d\rho^{\left(0\right)}}{|}_{\rho^{\left(0\right)}={\hat{\rho }}^{\left(0\right)}}\approx \frac{1}{H}\propto \frac{1}{\varepsilon }.$$

### Appendix B.3. Approximation of $\sum_{s}\psi \left(s\right)$

In primary creep, ε≪1 means that ε approaches 0 and the sequence of structure function approaches $\left\{s_{0}\right\}$. Therefore, Equation (12) is rewritten as

(A10) $$\sum_{s}\psi \left(s_{0}\right)\approx \left[1-h\left(\frac{s_{0}}{L}\right)\right]\prod_{i=0}^{s_{0}-1}h\left(\frac{i}{L}\right).$$

When the number of levels L≫1, it is further rewritten as

(A11) $$\begin{array}{ll} \left[1-h\left(\frac{s_{0}}{L}\right)\right]\prod_{i=0}^{s_{0}-1}h\left(\frac{i}{L}\right) & =\left[1-h\left(\frac{s_{0}}{L}\right)\right]\exp\left[\ln\;\prod_{i=0}^{s_{0}-1}h\left(\frac{i}{L}\right)\right]\\ & \approx \left[1-h\left(x_{0}\right)\right]\exp[L\int_{0}^{x_{0}}\ln\;h\left(x\right)dx] \end{array}.$$

When L≫1, $x_{0}=\frac{s_{0}}{L}\ll 1$, and Equation (A11) is simplified to

(A12) $$\exp\left[L\int_{0}^{x_{0}}\ln\;h\left(x\right)dx\right]\propto \exp\left[L\times \ln\;h\left(0\right)\times x_{0}\right]=\exp\left[\ln\;h\left(0\right)\times s_{0}\right].$$

Further simplification can be obtained based on Equation (A6) as

(A13) $$\sum_{s}\psi \left(s\right)\approx \exp\left[\ln\;h\left(0\right)\times s_{0}\right]\approx {\left(\varepsilon^{-\frac{1}{\ln\;a}}\right)}^{\ln\;h\left(0\right)}=\varepsilon^{-\log_{a}\;h\left(0\right)}.$$
